# Neuroprotective effects of berberine in animal models of Alzheimer’s disease: a systematic review of pre-clinical studies

**DOI:** 10.1186/s12906-019-2510-z

**Published:** 2019-05-23

**Authors:** Ning-Ning Yuan, Cui-Zan Cai, Ming-Yue Wu, Huan-Xing Su, Min Li, Jia-Hong Lu

**Affiliations:** 1 State Key Laboratory of Quality Research in Chinese Medicine, Institute of Chinese Medical Sciences, University of Macau, Taipa, Macao, Special Administrative Region of China; 2 Centre for Parkinson’s Disease Research, School of Chinese Medicine, Hong Kong Baptist University, Kowloon Tong, Hong Kong, Special Administrative Region of China

**Keywords:** Berberine, Alzheimer’s disease, Neuroprotection, Dementia, Animal models

## Abstract

**Background:**

Berberine is an isoquinoline alkaloid extracted from various *Berberis* species which is widely used in East Asia for a wide range of symptoms. Recently, neuroprotective effects of berberine in Alzheimer’s disease (AD) animal models are being extensively reported. So far, no clinical trial has been carried out on the neuroprotective effects of berberine. However, a review of the experimental data is needed before choosing berberine as a candidate drug for clinical experiments. We conducted a systematic review on AD rodent models to analyze the drug effects with minimal selection bias.

**Methods:**

Five online literature databases were searched to find publications reporting studies of the effect of berberine treatment on animal models of AD. Up to March 2018, 15 papers were identified to describe the efficacy of berberine.

**Results:**

The included 15 articles met our inclusion criteria with different quality ranging from 3 to 5. We analyzed data extracted from full texts with regard to pharmacological effects and potential anti-Alzheimer’s properties. Our analysis revealed that in multiple memory defects animal models, berberine showed significant memory-improving activities with multiple mechanisms, such as anti-inflammation, anti-oxidative stress, cholinesterase (ChE) inhibition and anti-amyloid effects.

**Conclusion:**

AD is likely to be a complex disease driven by multiple factors. Yet, many therapeutic strategies based on lowering β-amyloid have failed in clinical trials. This suggest that the threapy should not base on a single cause of Alzheimer’s disease but rather a number of different pathways that lead to the disease. Overall we think that berberine can be a promising multipotent agent to combat Alzheimer’s disease.

## Background

Alzheimer’s disease (AD) is a progressive degenerative disease of the central nervous system. Its main clinical manifestations are progressive declines in memory and cognitive function, accompanied by psychiatric symptoms and abnormal behavior. AD mostly occurs in elderly persons over 65 years of age. According to 2017 statistics, there are nearly 46 million AD patients worldwide [1, 2]. In the brain, senile plaques (SP) and neurofibrillary tangles (NFT) are the diagnostic hall markers of AD. Its other pathological features include diffuse atrophy of the cortex, widening of the sulcus, enlargement of the ventricles, loss of neurons and decreases in choline acetylase and acetylcholine levels. The etiology of AD is still elusive, and several hypotheses have been proposed to explain the pathogenesis of AD. The most prevalent hypotheses are the amyloid β-protein (Aβ) cascade hypothesis [3, 4], hyper-phosphorylated Tau hypothesis [4], the free radical theory [5], the inflammatory theory [6] and cholinergic hypothesis [7]. The diversity and uncertainty of the pathogenesis of AD have caused difficulties in the development of effective treatment, and most of the clinical trials performed in recent decades have failed.

**Fig. 1:**
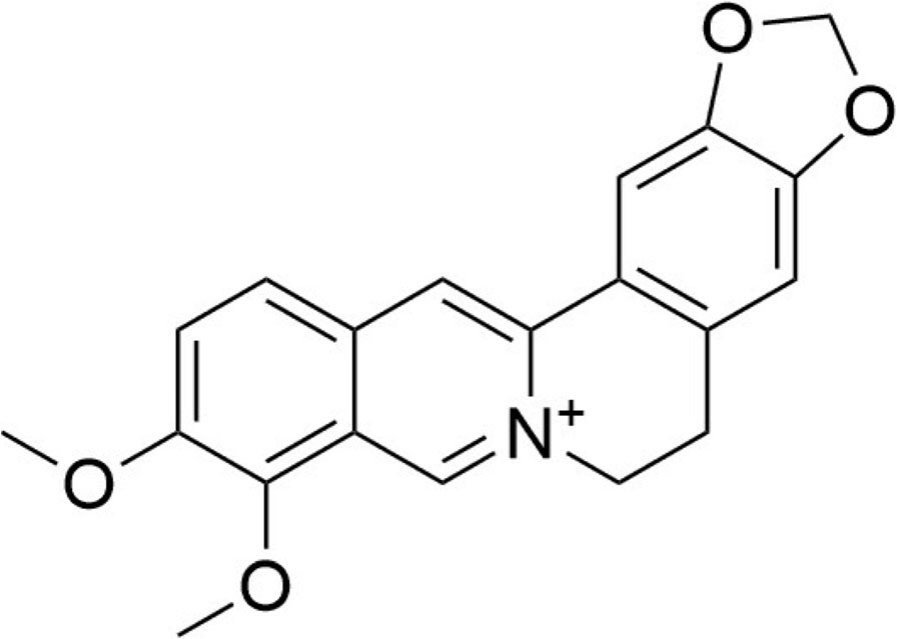
Chemical structures of berberine

Berberine is an isoquinoline alkaloid that is widely present in several medicinal plants, especially in those belonging to the *Berberis* genus (e.g., *Berberis vulgaris* L., Berberidaceae). It also occurs, for example, in *Coptis chinensis* Franch. (Ranunculaceae), a plant which is used in traditional Chinese medicine as an anti-diarrheal, anti-bacterial, anti-fungal, and anti-protozoal agent, particularly in combination with other herbs [8–10]. The chemical structure of berberine is shown in Fig. 1. In several years, accumulating evidence has revealed a wide variety of bioactivities of berberine such as antiviral, antibacterial and anti-inflammatory [11, 12].

The pharmacological effect of berberine on the nervous system was first reported in the 1970s as sedation-inducing [13]. The therapeutic activity of berberine has been widely examined in various neurological conditions including cerebral ischemic injury, AD, Parkinson’s disease, depression, anxiety, Huntington’s disease, epilepsy and convulsions. Several studies have shown that berberine can alleviate AD pathology through various mechanisms, including inhibition of hyper-phosphorylation of Tau protein and Aβ production. Berberine can reduce the hyper-phosphorylation of Tau protein, and this reduction may be related to the activation of the phosphatidylinositol 3-kinase/protein kinase/glycogen synthase kinase 3 pathway to restore protein phosphatase 2A activity and reverse glycogen synthase kinase-3 (GSK-3) activation [14]. In addition, berberine can inhibit the expression of beta-secretase by activating the extracellular signal-regulated kinase 1/2 signaling pathway, thereby inhibiting the production of Aβ40/42 [15]. Moreover, researchers have recently revealed that, on a molecular basis, berberine exerts inhibitory effects on the four key enzymes in the pathogenesis of AD: acetylcholinesterase, butyrylcholinesterase, monoamine oxidase A, and monoamine oxidase B [16].

Before this, several experiments have been performed to evaluate the anti-AD properties of berberine. However, these pre-clinical studies have not been systematically analyzed to provide a whole picture and un-biased understanding of the therapeutic potential of berberine for AD. The aim of this systematic review is to summarize the current evidence and analyze that evidence as to what it reveals about the underlying mechanism of the protective effects of berberine in animal AD models. We hope to provide more insightful information for future clinic trials.

**Fig. 2:**
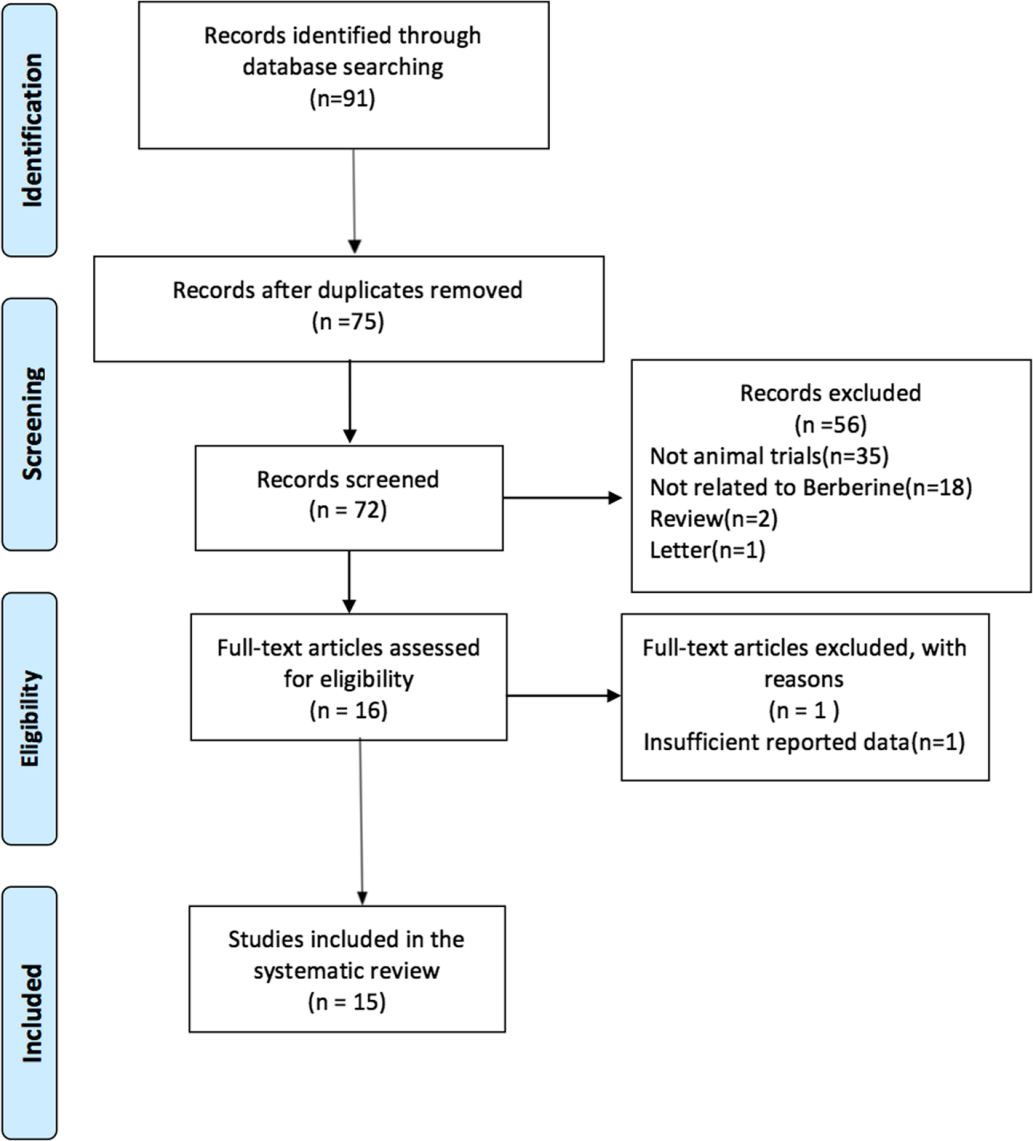
Research methodology for review process

To perform the systematic review, we searched the literatures and selected the studies passing our selection criteria for data extraction and analysis. Our search of electronic databases returned a total of 91 articles. After deleting 16 which contained duplicated experimental data, we had a total of 72 references. After reading the titles and abstracts, we deleted 57 papers for the following reasons: (1) Not including experiments on animal models; (2) Not directly administering berberine; (3) No experimental details provided. Thus, finally, we had 15 articles that reported the efficacy of berberine in AD animal models; this review is based on these articles (Fig. 2).

## Method

### Literature search

A careful literature search was performed to find publications reporting studies of the effect of berberine treatment on animal models of AD. Online literature databases (PubMed, Google scholar, PsychINFO, Embase and Web of Science) were searched up to March 2018 using search terms for English or Chinese publications. The following search strategy was used for each database.

1. Berberine
2. Alzheimer’s Disease
3. Alzheimer Disease
4. AD
5. or/2–4
6. 1 and 5

### Inclusion/exclusion criteria and screening

#### Inclusion criteria

1. Berberine was administered alone.
2. Experimental AD was induced in rodents (i.e., rats or mice).
3. AD treatment group was treated with a pharmacological agent, and a control group was administered a placebo after injury.
4. Article was published in English or Chinese.

#### Exclusion criteria

1. Not an original paper (review or letter etc.);
2. Berberine was not administered alone.
3. Absence of a correct control group.
4. Other types of animals (e.g., sheep, cats, and dogs) were used.
5. Duplicate publications.

### Data extraction and quality assessment

#### Data extraction

Two investigators independently screened papers and listed them based on publication year, the first author’s name and experimental models. Using a structured form, they extracted individual data on study characteristics, methods and outcome measures. The differences in papers selected were resolved through discussion. Finally, the methodological quality of the included basic research was assessed by applying six correction scales.

## Results

The search strategy retrieved 91 papers through online literature databases (PubMed, Google scholar, PsychINFO, Embase and Web of Science), 15 papers met our selection criteria. These 15 studies evaluated in this review involved animals from two species and four varieties: TgCRND8 mice, APP/PS1 mice, Sprague Dawley rats and Wister rats. The scales of the studies varied, from 6 to 104 animals in a single study. Rat and mouse weights were 200–300 g and 20–55 g, respectively. Eleven studies used male animals, and 1 study used female rats. After selecting and classifying these 15 studies, 3 were diabetic rat models with memory-impairment, 2 were 3 × Tg-AD mice models, 2 were Aβ infused rats models, 1 was an APP/PS1 mice model, 1 was a (Pilo)-induced epilepsy rat model, 1 was an ibotenic acid (ibo)-induced rat model and 5 memory-impairment models induced by Scopolamine, ICV-STZ, ethanol and D-galactose respectively. The research parameters evaluated in the 15 studies included the Morris water maze, immunohistochemistry (IHC), Western blot, RT-PCR (reverse transcription-polymerase chain reaction) and ELISA. The Morris water maze, a behavior test, was used to evaluate memory function. The IHC method as a molecular biology technique was used to investigate neuroprotective effects. Western blot, ELISA and RT-PCR techniques were used to measure potential genetic and proteins markers involved in Alzheimer’s disease. Table 1 lists the basic characteristics of the 15 studies.

**Table 1:** Basic information of included studies

Study	Animal Models	Administration(dosage, time and route)	Evaluation methods of the treatment effectiveness test
He et al. [17]	Thirty 120-day APP/PS1 mice (male, 30)	0, 50 and 100 mg/kg-berberine (*n* = 10 for each group), intragastric administration for 14 days.	MWM, WB analysis, immunohistochemistry
Chen et al. [18]	Aged 4–5 weeks Wistar rats weighting about 200 g memory-impairment diabetic Wistar Rats model (male)	DM group (diabetes mellitus), berberine group (187.75 mg/kg/d) and Met group (Metformin, 184 mg/kg/d), orally administration for 2 weeks.	Fear Condition, PET, WB analysis, immunochemistry
Huang et al. [19]	4-month 3 × Tg-AD (male,18;female,18)	0, 50 and 100 mg/kg berberine (*n* = 12 for each group), orally administration for 4 months.	MWM, platform recognition, HVS water maze, Aβ1–42: Elisa, WB analysis, Immunofluorescence staining, histological analysis
Oliveira et al. [20]	300–350 g ICV-STZ induce sporadic Alzheimer’s-like dementia Wistar rats model (male, 60)	Control (CTR), berberine 50 mg/kg, berberine 100 mg/kg, streptozotocin, streptozotocin plus berberine 50 mg/kg, and streptozotocin plus berberine 100 mg/kg, orally administration for 21 days.	MWM, Elevated plus maze task, LDH: Labtest kit, AChE activity
Patil et al. [21]	200–250 g memory-impairment Wistar rats model induced by ethanol (male,12)	25, 50 and 100 mg/kg berberine, vitamin C (100 mg/kg), and vehicle (1 mL/kg), DDW control rats(double distilled water), orally administration (chronic treatment: once a day for 45 days before training; acute treatment: once a day for 5 days during training).	MWM, Memory consolidation test, Elevated plus maze test, ChE activity
Haghani et al. [22]	200–250 g 60–65 days Aβ infused Wistar rats model (male, 32)	Sham(normal saline), berberine(50 mg/kg), Aβ(normal saline)and Aβ + berberine(berberine 50 mg/kg) (*n* = 8 rats in each group), intraperitoneal administration daily for 13 days.	MWM, Passive avoidance test,
Zhan et al. [23]	180–220 g, 10–12 weeks D-galactose-induced memory impairment Wistar rats (male)	Berberine (100 mg/kg per day), orally administration for 7 weeks.	MWM, WB analysis, RT-PCR
Gao et al. [24]	200–250 g (Pilo)-induced epilepsy Sprague Dawley rats (male,104)	1) control group (n = 12), saline; 2) berberine 100 mg/kg group (n = 12); 3) Pilo group (*n* = 20); 4) Pilo + berberine 25 mg/kg group (n = 20); 5) Pilo + berberine 50 mg/ kg group (n = 20); 6) Pilo + berberine 100 mg/ kg group (n = 20). Orally administration daily for 7 days before Pilo injection.	MWM, GSH level: sectrophotometrical
Moghaddam et al. [25]	(STZ)-diabetic Wistar rats (male,50)	Control, berberine-treated control (100 mg/kg;), diabetic, and berberine-treated diabetics (50 and 100 mg/kg respectively).	Y-maze, Single-trial passive avoidance test, Electrophysiological experiments
Durairajan et al. [15]	TgCRND8 mice	Berberine (25 mg/kg per day), berberine (100 mg/kg per day), orally administeration	MWM, ELISA, Immunohistochemistry, WB analyses
Lee et al. [26]	260–280 g SCO-induced memory deficits SD rats (male)	SAL group, PA group, SCO-induced and saline-treated group, SCO + berberine20 mg/kg group,SCO + PA100 group, SCO-induced plus 200 mg/kg PA-treated group, and the SCO-induced plus 0.2 mg/kg TA-treated group. Intraperitoneally administration once a day for 14 days.	MWM, Hidden platform trial, Probe trial, immunohistochemical, ELISA,
Bhutada et al. [27]	Memory-impairment diabetic Wistar rats (male)	1.berberine (25, 50, 100 mg/kg), vitamin C (100 mg/kg), metformin (500 mg/kg), or vehicle (1 mL/kg) twice daily for 30 days. Orally administration 2.berberine (25, 50, 100 mg/kg), vitamin C (100 mg/kg), metformin (500 mg/kg), vehicle (1 mL/kg), twice daily during training trials for next 5 days. Orally administration	MWM, Memory consolidation test, Open field test
Lim et al. [28]	Ibotenic acid-induced memory deficient Sprague Dawley rat model (male)	IBO model(saline), IBO model(berberine), saline injected sham group(berberine), daily Intraperitoneally administration for a week.	immunohistochemistry
Zhu et al. [29]	injected A-beta (1–40) (5 microgram) AD rat model	Berberine chloride (50 mg/kg), Orally administration once daily for 14 days.	MWM, immunohistochemistry, PCR
Peng et al. [30]	200–250 g SCOP-induced amnesia Sprague-Dawley rats (male)	Berberine (0.1 and 0.5 g/kg) orally daily for 7-day or 14-day.	Passive avoidance response task, Motor activity measurements

MWM, Morris water maze; WB analysis, Western Blot analysis; PET, Positron-Emission Tomography; RT-PCR, Reverse Transcription Polymerase Chain Reaction; AchE, Acethyl-cholinesterase; ICV-STZ, Intracerebroventricular streptozotocin; GSH, glutathione; Pilo, pilocarpine;

### Methodological quality

We assessed the scores of the quality according to these 6 points:

A: peer reviewed publication; B: random allocation to group; C: blinded assessment of outcome; D: a sample size calculation; E: compliance with animal welfare regulations; F: a statement of a potential conflict of interest.

The quality items scored in the included studies ranged from 3 to 5 out of a total of 6 points as shown in the Table 2. Two of the studies (13.3%) achieved 3 points; seven studies (46.7%) achieved 4 points; and Six studies (40%) achieved 5 points.

**Table 2:** Methodological quality of included studies

Study	A	B	C	D	E	F	Total
He et al. [20]	√	√		√	√	√	5
Chen et al. [27]	√	√			√	√	4
Huang et al. [21]	√	√		√	√	√	5
Oliveira et al. [19]	√	√		√	√	√	5
Patil et al. [17]	√	√		√	√		4
Haghani et al. [31]	√	√		√	√	√	5
Zhan et al. [32]	√	√	√		√		4
Gao et al. [22]	√	√		√	√	√	5
Moghaddam et al. [23]	√	√		√	√		4
Durairajan et al. [15]	√	√		√	√	√	5
Lee et al. [33]	√	√			√		3
Bhutada et al. [34]	√	√			√		3
Lim et al. [24]	√	√	√		√		4
Zhu et al. [25]	√	√	√		√		4
Peng et al. [28]	√	√	√		√		4

Table 2 shows the Methodological quality of the 15 reviewed studies.

### Anti-Alzheimer’s disease mechanisms of berberine

**Fig. 3:**
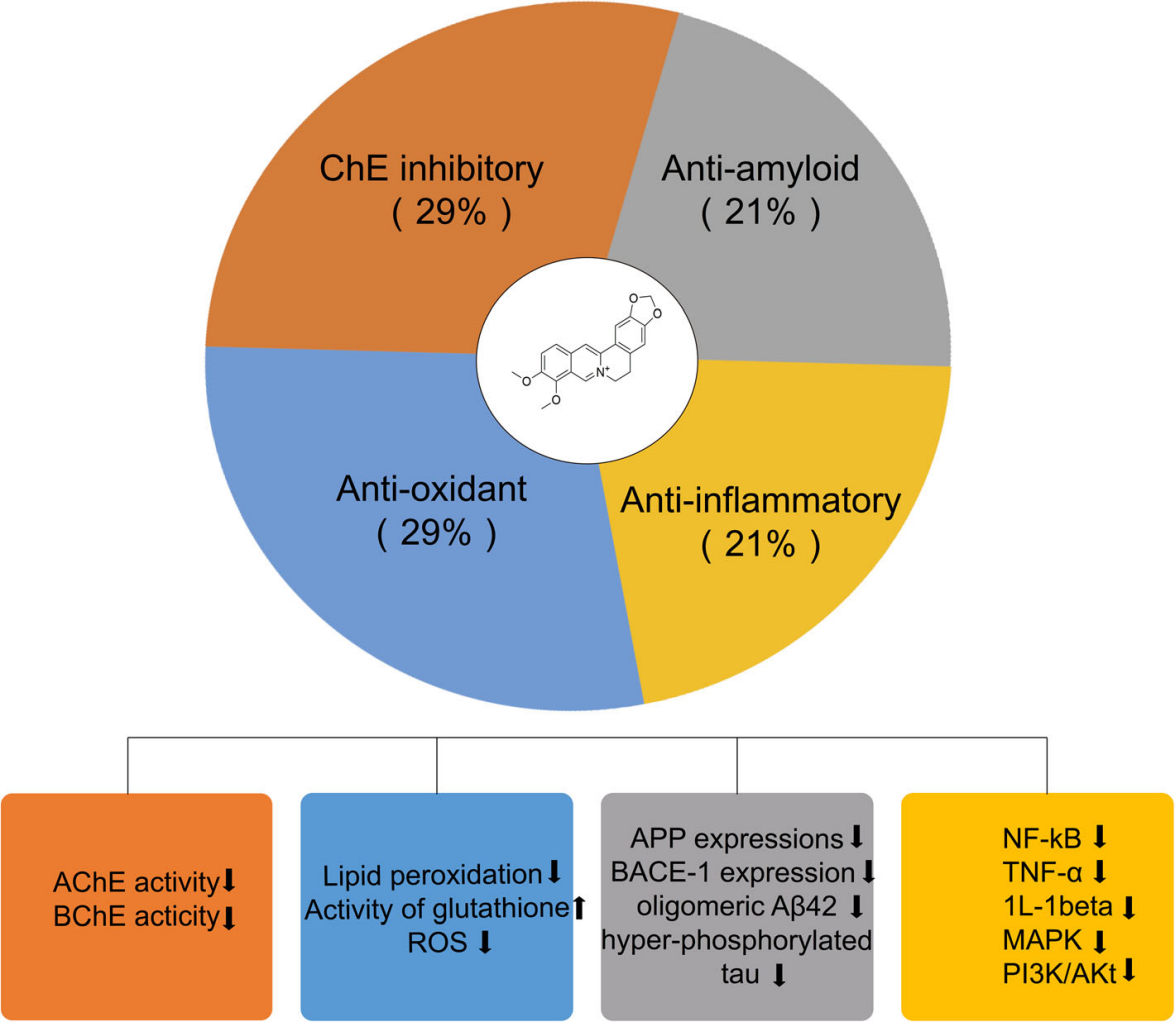
Reported potential mechanisms underlying anti-AD property of berberine

Table 3 shows the main outcomes and results of the included studies. Twelve studies investigated whether berberine improved cognitive abilities; four studies examined hippocampal cells of CA1 region and apoptosis of pyramidal neurons in the CA1 area. The changes in oxidative stress and acetylcholinesterase (AChE) activity were examined in 8 studies. Three studies tested NF-kB signaling. In addition, one study reported that berberine induced autophagy to reduce the APP and BACE1 levels. The above proposed neuroprotective mechanisms of berberine are summarized in Fig. 3.

**Table 3:** Anti-AD effects and underlying mechanisms after berberine treatment of included studies

Study	Anti-AD effects	Neuroprotection mechanism
He et al. [17]	1. Mitigated cognitive impairment of AD mice. 2. Inhibited phosphorylation of Tau. 3. Limited lipid peroxidation. 4. Lowered the levels of IL-1b and TNF-a. 5. Lowered the levels of both GFAP and CD45.	Anti-inflammatory, suppressed NF-kB signaling pathway, anti-oxidative stress and anti-apoptosis.
Chen et al. [18]	1. Inhibited the inflammation mediator release and insulin resistance in the mPFC of diabetic rats.2. Ameliorated cognitive impairment and accelerates the reinforcement of the information. 3. Decreased the expressions of amyloid precursor protein and BACE-1, and the production of oligomeric Aβ42.	Inhibits the PI3K/Akt/mTOR and MAPK signaling pathway, as well as two novel isoforms PKCη and PKCε and the translocation of NF-κB Anti-inflammatory. Anti-amyloid.
Huang et al. [19]	1. Ameliorated cognitive deficits, improved spatial learning capacity and memory retention in 3 × Tg-AD mice model2. Reduced the production of Aβ and BACE1 protein level in primary hippocampal neurons and the brains of 3 × Tg-AD mice	Anti-amyloid, enhancing autophagy through the class III PI3K/beclin-1 pathway. Anti-apoptosis.
Oliveira et al. [20]	1. Prevented the memory loss, anxiogenic behavior 2. Reduced escape latency in ICV-STZ rats 3. Reduced the number of dead cells in both the hippocampus and cerebral cortex in STZ rat. 4. Decreased AChE activity in both the hippocampus and cerebral cortex of ICV-STZ rat.	ChE inhibition.
Patil et al. [21]	1. Improved ethanol-induced memory impairment. 2. Lowered oxidative stress and ChE activity in ethanol treated rats.	Anti-oxidant and ChE inhibition.
Haghani et al. [22]	1. Prevented the impairing impacts of Aβ on the learning, memory and electrophysiological properties of the CA1 pyramidal neurons. 2. Improved the memory performance. 3. Restored the Aβ-induced impairments in the firing frequency, half-width and rebound action potential.	Phenomenon research.
Zhan et al. [23]	1. Rescued D-galactose-induced memory impairment the mRNA and protein levels of Arc/Arg3.12. Reversed the synaptic deficits induced by D-galactose.	Phenomenon research.
Gao et al. [24]	1. Relieved pilocarpine-induced convulsions in rats. 2. Reduced the degree of oxidative stress in the hippocampus.3. Attenuated memory impairment. 4. alleviated neuronal degeneration in hippocampal CA1 region in SE rats.	Anti-oxidant.
Moghaddam et al. [25]	1. Ameliorated learning and memory impairment.2. Restored PS amplitude and fEPSP and ameliorated learning and memory impairment and attenuated apoptosis of pyramidal neurons in the CA1 area.	Anti-apoptosis.
Durairajan et al. [15]	1. Lower Aβ levels, alleviated cognitive deficits and amyloid neuropathology, reduce gliosis. 2. Reduced the Aβ and CTFs, probably by downregulating the phosphorylation of APP and of CTFs via the activation of the PI3K/Akt/GSK3 pathway. 3. Reduced the cognitive impairment 4. Decreased Aβ plaques of all 3 size subsets (25, 25–50, and > 50 μm). 5. Reduced vascular amyloids as well as parenchymal amyloids 6. Reducing ThioS-positive vascular amyloids. 7. 45% reduction in microgliosis and a 54% decrease in astrocytosis	Anti-amyloid, activation of the PI3K/Akt/GSK3 pathway
Lee et al. [26]	1. Improved memory impairment and reduced the escape latency.2. Alleviated memory-associated decreases and restored brain-derived neurotrophic factor and cAMP-response element-binding protein mRNA expression in the hippocampus.3. Decreases the expression of proinflammatory cytokines mRNA in the hippocampus.	Anti-inflammatory.
Bhutada et al. [27]	1. Improved cognitive performance. 2. Lowered hyperglycemia, oxidative stress, and ChE activity in diabetic rats.	Anti-oxidant. ChE inhibition.
Lim et al. [28]	1. Increased neuronal cells immunoreactive to calbindin in the hippocampus and entorhinal cortex area. 2. Hippocampal cells were increased in the pyramidal layer of CA1 region and dentate gyrus	Phenomenon research.
Zhu et al. [29]	Ameliorates the spatial memory impairment.	Phenomenon research.
Peng et al. [30]	Improved SCO-induced amnesia	Phenomenon research.

ChE, cholinesterase; IL-1β, Interleukin 1 beta; TNF-α, Tumor necrosis factor alpha; GFAP, Glial fibrillary acidic protein; CD45, CD45 Antigen; mPFC, The medial prefrontal cortex; BACE-1, β-site amyloid precursor protein cleaving enzyme 1; ICV-STZ, Intracerebroventricular streptozotocin; NF-kB, Nuclear factor kappa-light-chain-enhancer of activated B cells; MAPK, Mitogen-activated protein kinases; PKCη, Protein kinase C-eta type; PKCε, Protein kinase C epsilon type; fEPSP, Field excitatory postsynaptic potential; cAMP, Cyclic adenosine monophosphate

## Discussion

### Potential mechanisms underlying anti-Alzheimer’s disease properties of berberine

The neuroprotective effects of berberine have been extensively studied in different animal experimental models and we summarized the studies which include a rat model of amyloid beta induced-Alzheimer’s disease, a memory impairment model induced by ethanol in rats, a D-galactose-induced memory deficits model in rats, a pilocarpine (Pilo)-induced epilepsy model in rats, a scopolamine and streptozotocin-induced memory impairment model in rats, a memory-deficient rat model induced by stereotaxic injection of ibotenic acid into entorhinal cortex (Ibo model), and the transgenic mouse model of Alzheimer’s disease. Interestingly, berberine displayed significant effects in preventing memory impairment in these mechanistically different animal models, suggesting an over-all improvement of memory function by berberine. Indeed, mechanistic studies showed that berberine modulated a wide range of biological functions to exert neuroprotection and the detailed mechanisms are discussed in the following part.

- Antioxidant properties of berberine

Alzheimer’s disease is characterized by extensive evidence of oxidative stress which is the result of uncontrolled production of reactive oxygen species (ROS) [35]. ROS has been regarded as a critical factor in the neuron dysfunction or death of neuronal cells that contribute to the pathogenesis of the disease [36]. Under normal conditions, the damage caused by oxygen free radicals can be controlled through a series of reactive antioxidant systems. However, under pathological conditions, the balance between oxidants and antioxidants is disturbed such that active oxygen production exceeds cellular antioxidant defenses. The antioxidant activity of berberine has been widely demonstrated [34, 37–39]. For instance, berberine displayed peroxynitrite (ONOO^−^) scavenging activity and total ROS inhibitory capacities [37]. Bhutada et al. [27] showed that berberine treatment during training trials also improved learning and memory, lowered hyperglycemia, oxidative stress, and ChE activity in diabetic rats.

(b) Anti-inflammatory properties of berberine

In the brain of patients with Alzheimer’s disease, chronic inflammation has been well described. On the histological level, this inflammation is characterized by activated microglia, reactive astrocytes and increased inflammatory cytokines release [33]. This observation has led to the hypothesis that brain inflammation is a cause of neuronal damage in AD and anti-inflammatory drugs may be used as protective agents. Chen et al. [18] studied the functions of berberine involved in anti-inflammation and the amelioration of insulin resistance in the prefrontal cortex of diabetic rats. They found that intragastric administration of berberine (187.5 mg/kg/d) inhibited inflammation mediator release and insulin resistance in the mPFC of diabetic rats. Finally, it relieved the impairment of cognitive function in diabetic rats. The promising effect of *Phellodendron amurense* (PA) and its major alkaloid compound, berberine, on memory dysfunction has also been studied in scopolamine-induced memory deficient rats [26]. A two-week administration of 20 mg/ kg of berberine improved memory impairment as measured by the passive avoidance test, and it reduced the escape latency for finding the platform in the Morris water maze test.

(c) Anti-cholinesterase activity of berberine

The cholinergic hypothesis was initially presented several years ago, then several studies demonstrated the adverse effects of anticholinergic drugs on memory [40], the low intracerebral cholinergic activity in patients with Alzheimer’s disease (AD) [41, 42] and the association of AD with cholinergic transmission disorders [43]. This hypothesis suggests that decreased cholinergic activity is associated with the AD symptoms and the improvement of cholinergic activity will relieve the AD symptoms. The cholinesterase (ChE) is the major enzyme for acetylcholine destruction and its inhibition results in increasing acetylcholine level in the brain. Therefore, many anti-AD pharmacological studies have focused on cholinesterase (ChE) inhibitors to ameliorate the cognitive symptoms [44]. Several studies have been performed to examine the effect of berberine on the ChE activity. For example, chronic treatment with berberine (25–100 mg/kg) lowered oxidative stress and ChE activity in ethanol treated rats [21]. A similar promising effect of one-month treatment with berberine on streptozotocin-induced memory impairment in rats has been reported [20]. In another set of experiments, berberine (100 mg/kg) treatment during training trials also improved learning and memory and lowered hyperglycemia, oxidative stress, and ChE activity [27].

(d) Anti-amyloid activity of berberine

The 42-amino acid amyloid beta (Aβ) is released from cleavage of the amyloid precursor protein by β-secretase and γ-secretase [45]. The Aβ sequenced from the meningeal blood vessels of AD patients and individuals with Downs’ syndrome is highly aggregated, and spontaneously assumes the β-sheet conformation and polymerizes into oligomers, fibrils, fibrils and plaques [46]. Berberine has been shown to ameliorates β-amyloid pathology and cognitive impairment in an AD transgenic mouse model [19]. After berberine treatment, the levels of extracellular and intracellular Aβ1–42 were decreased, mediated by increased autophagy activity.

With advances in science, there is increasing interest in another constituent of neurofibrillary tangles(NFTs), hyper-phosphorylated Tau protein. He et al. found that berberine improved learning and memory in APP/PS1 mice, decreased hyper-phosphorylated Tau protein and lowered the activity of NF-kB signaling in the hippocampus of APP/PS1 mice [17]. Berberine administration promoted the activity of glutathione (GSH) and inhibited lipid peroxidation in the hippocampus of AD mice. They concluded that berberine attenuated cognitive deficits and limited hyper-phosphorylation of Tau via inhibiting the activation of the NF-kB signaling pathway and by retarding oxidative stress and neuro-inflammation.

### Opportunities and challenges

Berberine is a natural product with a definite structure and a wide range of pharmacological effects. Berberine displays many biological functions and potential therapeutic applications in neurological diseases. Animal research is an essential early step toward evaluating and developing an intervention for clinical trials in humans [31]. This systematic review has examined high quality animal studies on the anti-AD effects of berberine and finds a consistent effect of berberine in improving the memory defects in multiple animal models, indicating the therapeutic potential of berberine for treating AD. While the effects are clear, the mechanism is not; further research is needed to determine the details of the biochemical mechanisms and specific drug target(s). Meanwhile, perhaps the greatest barrier to the pharmaceutical development of berberine is its naturally low bioavailability. More effort, for example, in structural modification and/or pharmaceutical processing, is needed for berberine to achieve its full potential in clinical use [32]. The following suggestions are worth considering: 1. The feasibility of targeted drug delivery should be explored. It is difficult to achieve effective concentrations, especially in the brain, by oral administration so targeted administration is worth considering; 2. The effects of berberine in combination with other drugs for AD treatment can be tested. 3. The possibility of toxic effects of berberine during long-term drug administration must be considered, and thoroughly studied.

## Conclusions

In this paper, we have reviewed 15 high-quality animal studies on the neuroprotective effects of berberine against AD, with systematic evaluation of its efficacy and pharmacology mechanisms. Berberine has showed significant memory-improving activities in multiple memory defects animal models; common properties, including anti-oxidation, anti-inflammation and anti-ChE activity were revealed. So far, no clinical trial has been carried out on the neuroprotective effects of berberine. Considering the positive results from animal studies and the relatively low toxicity of berberine, the performance of clinical trials to evaluate the anti-AD effect of berberine on human patients appears justified.

## Abbreviations

AchE: Acethyl-cholinesterase
AD: Alzheimer’s disease
BACE-1: β-site amyloid precursor protein cleaving enzyme 1
BChE: Butyrylcholinesterase
cAMP: Cyclic adenosine monophosphate
CD45: CD45 Antigen
fEPSP: Field excitatory postsynaptic potential
GFAP: Glial fibrillary acidic protein
ICV-STZ: Intracerebroventricular streptozotocin
IL-1β: Interleukin 1 beta
MAPK: Mitogen-activated protein kinases
mPFC: The medial prefrontal cortex
MWM: Morris water maze
NF-kB: Nuclear factor kappa-light-chain-enhancer of activated B cells
PET: Positron-Emission Tomography
PKCε: Protein kinase C epsilon type
PKCη: Protein kinase C-eta type
RT-PCR: Reverse Transcription Polymerase Chain Reaction
TNF-α: Tumor necrosis factor alpha
WB analysis: Western Blot analysis
